# Palliative care and new technologies. The use of smart sensor technologies and its impact on the Total Care principle

**DOI:** 10.1186/s12904-023-01174-9

**Published:** 2023-04-26

**Authors:** Tabea Ott, Maria Heckel, Natalie Öhl, Tobias Steigleder, Nils C. Albrecht, Christoph Ostgathe, Peter Dabrock

**Affiliations:** 1 Chair of Systematic Theology II (Ethics), Faculty of Humanities, Social Sciences, and Theology, Friedrich-Alexander-Universität Erlangen-Nürnberg, Kochstraße 6, Erlangen, 91054 Germany; 2 Department of Palliative Medicine, University Hospital Erlangen, Friedrich-Alexander-Universität Erlangen-Nürnberg, Werner-von-Siemens-Straße 34, Erlangen, 91052 Germany; 3 Institute for High Frequency Technology, Hamburg University of Technology, Denickestraße 22 (I), 21073 Hamburg, Germany

**Keywords:** Total Care, Smart Sensor Technologies, Artificial Intelligence, Ethics, Quality of life

## Abstract

**Background:**

Palliative care is an integral part of health care, which in term has become increasingly technologized in recent decades. Lately, innovative smart sensors combined with artificial intelligence promise better diagnosis and treatment. But to date, it is unclear: how are palliative care concepts and their underlying assumptions about humans challenged by smart sensor technologies (SST) and how can care benefit from SST?

**Aims:**

The paper aims to identify changes and challenges in palliative care due to the use of SST. In addition, normative guiding criteria for the use of SST are developed.

**Methods:**

The principle of Total Care used by the European Association for Palliative Care (EAPC) forms the basis for the ethical analysis. Drawing on this, its underlying conceptions of the human and its socio-ethical aspects are examined with a phenomenological focus. In the second step, the advantages, limitations, and socio-ethical challenges of using SST with respect to the Total Care principle are explored. Finally, ethical-normative requirements for the application of SST are derived.

**Results and Conclusion:**

First, SST are limited in their measurement capabilities. Second, SST have an impact on human agency and autonomy. This concerns both the patient and the caregiver. Third, some aspects of the Total Care principle are likely to be marginalized due to the use of SST. The paper formulates normative requirements for using SST to serve human flourishing. It unfolds three criteria according to which SST must be aligned: (1) evidence and purposefulness, (2) autonomy, and (3) Total Care.

## Palliative care

The use of smart sensor technologies (SST) in healthcare, including palliative care, has the potential to revolutionize the way in which patients are diagnosed and treated. This paper inquires the opportunities and challenges associated with the use of SST in palliative care, particularly in terms of the impact on the underlying assumptions about humans and the principles of care that underpin this field. This results in three normative requirements according to which the use of SST must be aligned.

**Fig. 1:**
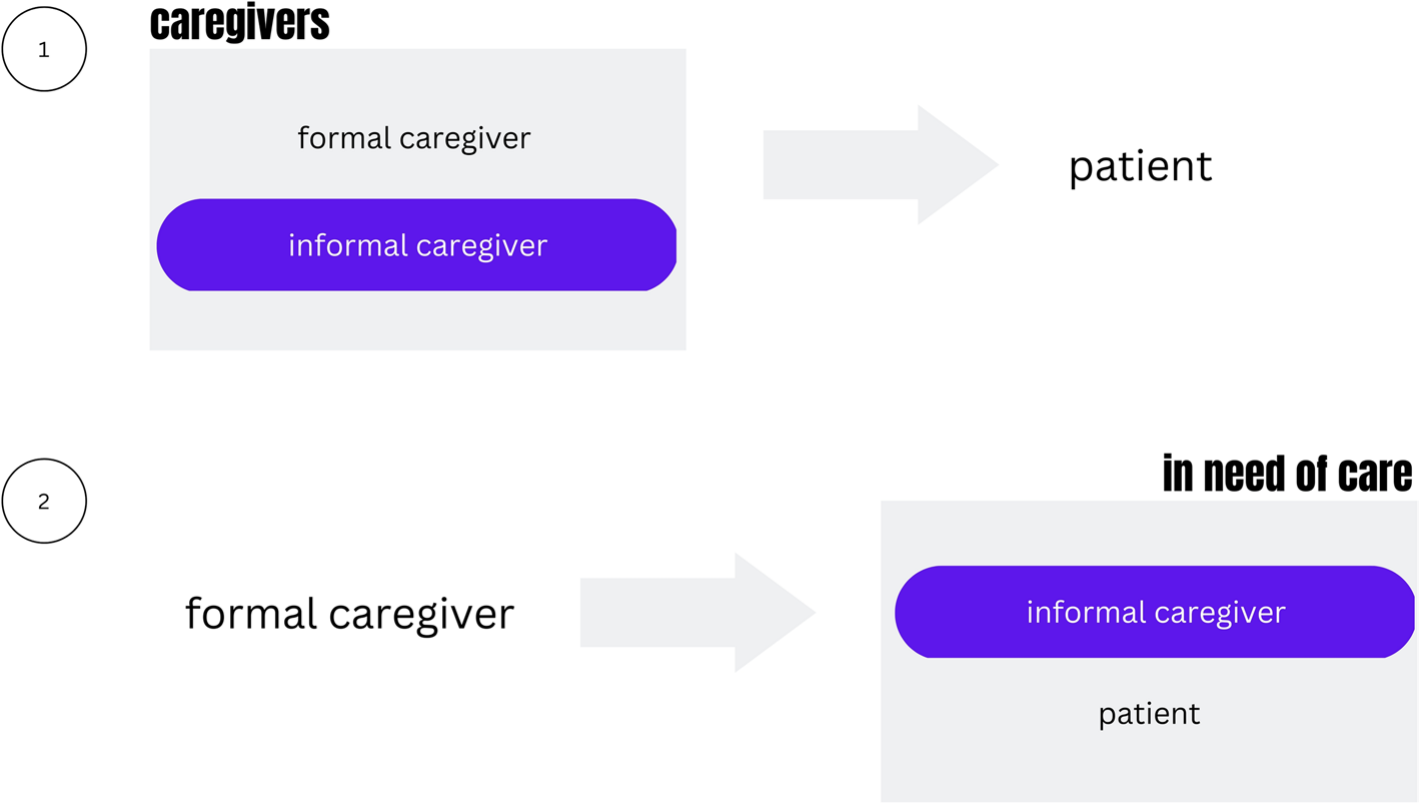
The situation of the informal caregiver

Palliative care focuses on improving the quality of life for patients who face a life-threatening illness. Several parts of this definition need explanation. First, quality of life is a highly subjective concept, which does not allow an objective assessment from the outside [1–4]. It depends on the combined effect of various, not only physical, factors. Second, the primary goal of treatment is palliation, not cure. Palliative care can be provided at all stages of the disease trajectory in parallel to curative treatment [4]. However, it is applied mainly in the phase after the diagnosis of the incurability of the underlying disease. Third, palliative care takes place in various settings: For many centuries, care for the very ill and the dying was provided by family members at home [5]. In palliative care, this private care is more and more embedded in public institutions – e.g. nursing homes, hospices, or hospitals [6]. In Germany, however, around 89% of people still wish to be cared for at home and in the United States, the number of people dying at home increased by 30,7% from 2003 to 2017 [7, 8]. With the rise of digital technologies in health care, services increasingly advertise combining the standard of clinical care with the comfort of one's own home by establishing “Home Health Care Centers” [9]. Fourth, palliative care faces complex supply situations, where the informal caregivers (family, friends, relatives, neighbors) have a dual role in which they are particularly vulnerable: they are caregivers (1) and those in need of care (2) at the same time – cf. Fig. 1 [10, 11].

Often, taking on care is accompanied by a change in role and relationship between informal caregivers and patients: Children, for instance, become deputies for their parents who are losing more and more of their ability to act independently [12, 13]. Additionally, the relationship between the dying patient and the team of caregivers is characterized by a difference in experience. The difference arises from the concrete experience of dying. It is the unique experience of the palliative patient which leads to the disappearance of the shared world of meaning between the patient and caregiver [14]. Martin W. Schnell even goes so far as to claim that empathy between participants is no longer possible [14]. Instead, it is essential to use “bridges” that the patient offers on his or her own accord [14]. Gadamer, too, argues that a common horizon is necessary for an understanding of the other – also in the sense of understanding as speaking for an Other in front of others [15, 16].

To conclude, “quality of life” is (1) not easy to determine. It has to be constantly redefined in (2) the changing life situation of a terminally ill person and (3) her care environment, which involves (4) complex care relationships. The experience of *total pain *[17] in the end-of-life phase requires, as a response, multidimensional and comprehensive care, referred to here as the *Total Care* principle. It provides a paradigm to account for a person’s responsiveness [18], including their subjectivity, complexity, changeability, relationality, and embeddedness. The Total Care principle provides the basis of a normative framework for the successful care of suffering and dying patients who are confronted with a loss of agency and an increase in dependency [19].

## Total care as principle of palliative care

**Fig. 2:**
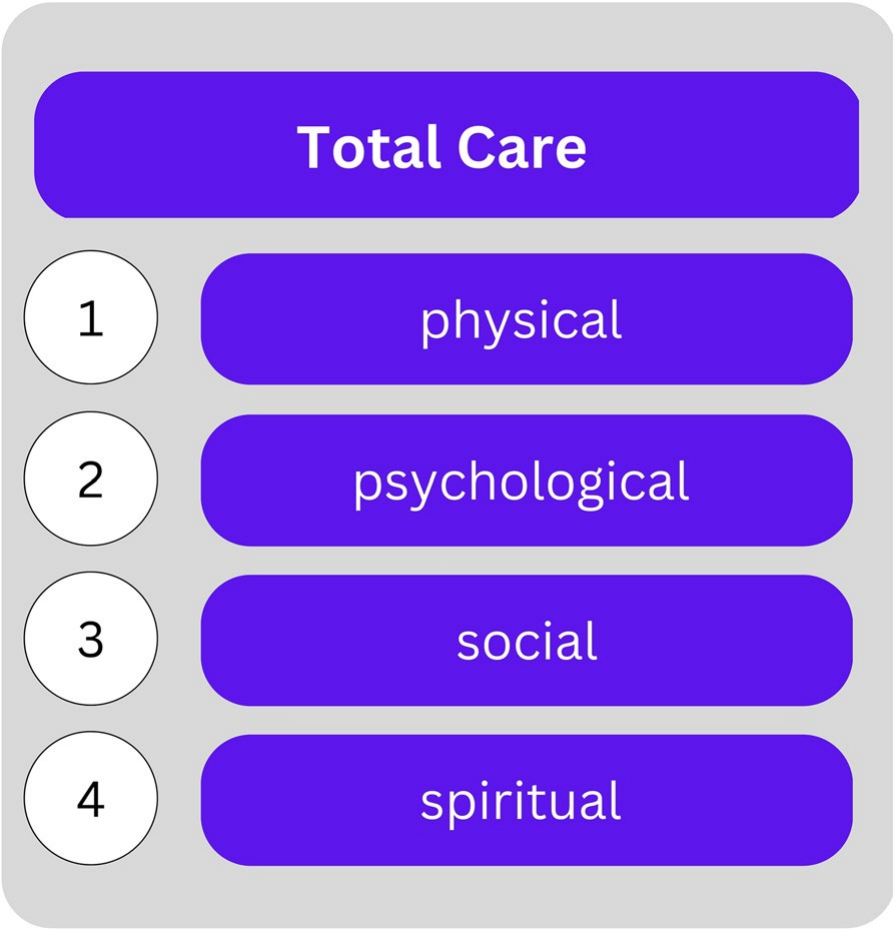
The Total Care principle

The European Association for Palliative Care (EAPC) uses the term **Total Care** in their recommendations 2009 to define standards of palliative care [20]. The principle of Total Care is used in response to the Total Pain concept of Cicely Saunders, who is considered one of the founders of modern hospice and palliative care [21]. According to Saunders, Total Pain describes pain not only as a physical symptom but a complex phenomenon that includes mental distress as well as social and spiritual suffering [17, 22]. This insight led her and others following her to a holistic concept of care that focuses on the person as an integral entity while incorporating the findings of humanities [23]. In particular, four dimensions are addressed by the Total Pain paradigm and the derived principle of Total Care: the physical (1), the psychological (2), the social (3), and the spiritual dimension (4) – cf. Fig. 2 [21]. These dimensions are not distinct from each other but are complexly intertwined, even if the outline of this paper suggests otherwise. Structuring them into separate dimensions serves to clarify them individually. At the same time, their interconnectedness will be emphasized for each.

Underlying the Total Care principle and its four dimensions are certain basic assumptions about human beings: (1) Humans are embodied. In German, a distinction can be made between ‘*Leib*’ (embodiment) and ‘*Körper*’ (body): Embodiment comprises the lifeworldly, i.e., individually and socially always interpreted body, which is more and reaches further than the intention of a mere objective corporeality. The embodiment is biologically and socially the anchoring of the ego in the world, the “zero point of orientation”[24] in the world. It has an epistemic peculiarity in the sense that the embodiment is always “*mitgegeben” *[25] (*given along*) in every encounter with the world [26]. The embodiment experiences and is experienced. It is a medium to the world and simultaneously itself a human subject. In illness, addiction, ageing, and sexuality, the I wishing for sovereignty experiences the embodiment – an experience that is no other than its own [27]. The body-phenomenological tradition has shown in connection with developmental-psychological, sociological and psychoanalytical insights that we as persons always already possess an interpreted, indirect access to the world – and responsively to ourselves. We never overcome this way – not even in our abstractions, in which we putatively refrain from it as it corresponds to the epistemological ideal of the natural sciences. Additionally, clothes that are (or are not) tailored to our bodies, body carvings, heart catheters, eyeglass frames, and drug use each show in their own way how the embodiment transcends mere corporeality, both internally and externally. Because of these complex interactions of inside and outside, of the social and the individual, of the cultural and the natural, corporeality can be interpreted, with Bernhard Waldenfels, as “*Zwischenleiblichkeit*” [18]. In our body, we are a crossing point of many biological imprintings as well as socio-cultural traditions. We ourselves continue to weave biologically and socially in this web of life. In this genealogically and systematically irrevocable in-between the experiences of I, you, third, we, your, you are constituted. Thus, the embodiment is embedded and formed in intersocial relations [28]. This interpretation of the human embodiment corresponds with the second underlying assumption of the Total Care principle: (2) Humans are relational and responsive beings. Their relationality influences their being and well-being [29]. Where the curing or the control of the disease no longer holds the driver’s seat in treatment planning and the quality of life is prioritized, this interconnectedness of the human comes more and more into view.

## How do smart sensor technologies affect the Total Care principle?

How is the principle of Total Care, which is not only but also in a unique way expressed in palliative care, challenged and supported by the use of smart sensor technologies (SST)? SST include all non or minimally invasive sensor technology aimed at the comprehensive collection of physical data – e.g., sweat, blood pressure, movements, and heart / respiratory rate [30, 31]. It is called smart because instead of conventional algorithms, machine learning is used to evaluate the data. The aim of applying these new technologies is – among others – to gain new insights into correlations between measurable parameters (respiratory rate, facial expression, etc.) and a person’s inner experience – or, to put it another way: to gain a more comprehensive understanding of a person’s general condition. At first glance, this aim seems promising regarding the Total Care principle. To achieve the best possible quality of life, symptom assessment and management play a pivotal role in palliative care. However, to date, assessment by health care professionals is discontinuous and only a snapshot of the clinical situation. Standard monitoring technologies are touch-based (e.g., ECG with its cables). They can irritate the patient and have a negative impact on mobility. Visible medical technology interferes with personal care and social inclusion. Relatives, for example, may be reluctant to touch or hug the patient. Thus, standard technologies give a continuous insight into the patient’s state of health but are a burden to the patient and not adequate in a palliative care setting [31]. As SST are non or minimally invasive in design, they offer undisturbed mobility for patients and prevent physical restrictions that come along with invasive technologies. In contrast to discontinuous assessment by health care professionals, SST monitor continuously (24/7). Hence, they can provide long-term data – without burdening the patient. These characteristics are particularly promising in palliative care settings, which are designed to be person-centered and as non-invasive and comfortable as possible. This kind of technology – possibly in combination with other digital applications – will shape future health and palliative care [32]. The purpose of this paper is to identify the normative foundations for the use of SST that must be given for the Total Care principle to be met. First, the paper problematizes the limitedness of quantifiable data and their absoluteness. Second, it asks how SST can support the individual patient’s autonomy, articulation, and changing wishes in difficult end-of-life situations. Finally, it questions whether SST can meet every aspect of the Total Care principle. The aim is to show that the use of SST, often referred to as “high tech”, and the human corporeality, their dependence on relationality in care settings, often referred to as “high touch”, is not mutually exclusive but can enrich each other [32, 33].

### Smart sensor technologies and the physical dimension of care

Patients with advanced disease suffer from a variety of physical symptoms – on average, 10 to 12 symptoms simultaneously [34, 35]. Finding their causes is crucial for targeted therapy and improving the quality of life [34]. However, palliative care deals with a high number of patients who do not report all symptoms [34]. This is caused by various factors. Claudia Bausewein et al. name as reasons resignation or the avoidance of being a burden to others. Another important factor is the reduced ability to speak and articulate due to the progression of a terminal disease, [36] accompanying diseases like dementia, [37] or the use of sedative drugs [38].

Regarding the identification of physical symptoms, SST promise great help. Sensors that measure, e.g., heartbeat precisely, enable calculating heart rate variability as a potential marker for the balance of the autonomic nervous system. They can help to determine stress due to pain or other symptoms. Additionally, these parameters may be used as indirect markers for the effectiveness of measures for symptom management, e.g., if HRV increases as a sign of less stress after a pain medication. Thus, SST can function as a kind of articulation aid or advocate. However, it must be taken into account that symptom experience (for example, pain, breathlessness, nausea) is profoundly individual and complex. The interpretation of data produced with SST cannot be considered in isolation. To contribute to the safeguarding of patient autonomy, other sources of guaranteeing autonomy or better “assisted freedom” [39] must be considered additionally – e. g. direct patient communication: the personal address is important, even for comatose or sedated patients, as well as the involvement of relatives or legal representatives. A risk lies in setting data from biomarkers as absolute and therein becoming paternalistic. Setting data as absolute negates first the fallibility of the measurement instruments (this can be due to by-catch, bias in the application of AI, or other factors) and their non-objectivity regarding the translation process of data into diagnosis or medical intervention [40–42]. Secondly, it runs the risk of neglecting the more holistic approach of the Total Care principle, which includes dimensions other than the physical that are not as easily or not at all measurable with SST. For example, it is likely to focus on “’easy-on-see’ physical aspects such as wound management, with less attention on psychosocial and spiritual aspects of care” [32]. Sheila Payne et al. clearly see a possible impact on the patient-professional relationship “that focuses on the data generated rather than holistic concerns of the patient” [43]. Ultimately, it must not be forgotten that the measured data are very likely to influence the patient’s own body experience, as studies with healthy subjects have demonstrated [44, 45].

### Smart sensor technologies and the psychological dimension of care

Closely related to the diagnosis and physical status of the patient is the psychological suffering. Here the body as Leib becomes relevant again: On the one hand, one’s own body interpretation and perception affect one’s own self-concept, one’s interpretation of oneself and the world. The situation of the body clouds the perception of the world. The experience of physical pain interrelates with the psychological dimension and vice versa [46]. Additionally, factors like social participation, which can become fragile in the course of a terminal illness, influence mental health [47]. In palliative care, a significant number of patients, as well as their relatives, show clinically relevant symptoms of psychological distress [34]. Within the first year after diagnosis, about half of the palliative patients develop a clinically diagnosable mental disorder [34].

As stated before, SST aim to gain a more comprehensive understanding of a person’s general condition by drawing conclusions from non-invasive measured body parameters to inner psychological states. The increase in the problem of defining the relevant aspects becomes evident when considering the psychological dimension and converting them into measurable parameters. Usually, besides the conversation between the patient and the health care professional, questionnaires and self-descriptions are used for standardized psychological assessments, for example, the Beck Anxiety Inventory (BAI) consisting of 21 self-reported items. The goal of SST is to support self-assessment by measuring external body responses that provide inferences about internal states. Concerning emotions – besides heart rate variability, respiratory rate, sweating, or gestures/postures of the body and mimics may be used as possible measurable markers [48, 49]. However, the visible and measurable body reaction sometimes differs from the self-report. To state their case, Mark Purdy et al. refer to an experiment by Paul Zak, who showed that for Super Bowl commercials, there are discrepancies between what people say and how they subconsciously feel or are emotionally involved [50]. By generalizing this finding with the quote, “People lie, their brains don’t” [50] and viewing SST as something that brings the truth to light, SST may become a paternalistic tool. The results and recommendations of SST would be set absolute, thereby endangering human autonomy and self-determination. Additionally, emotions and physical states are extremely subjective and culturally dependent, making the SST prone to bias [50].

### Smart sensor technologies and the social dimension of care

The third dimension of Total Care, the social dimension, addresses the connectedness of humans and their net of relationships. It considers the socially embedded embodiment of a patient. In the course of a terminal disease, there often is a major change in the circumstances of life. This change is not seldomly accompanied by loss of contacts, the experience of alienation from one’s own home and identity, as well as financial concerns [34]. However, within these changes, social interactions are of great importance for patients and their emotional well-being. SST can affect interactions and change the roles, tasks, and self-understanding of patients and caregivers. As SST deliver additional information on the condition of patients, the roles of patients and caregivers change, and they are no longer the only source of information on their well-being. However, they are no less important as a source of information. Additionally, the social dimension of Total Care considers the role of the informal caregivers and their care needs – these are the relatives, family, neighbors, and friends who have the double-edged role of those who are caregivers and due to their physical and emotional involvement in need of care at the same time [11, 51]. The impact of the SST’s collection of patient data on informal caregivers is twofold: On the one hand, the collected data may allow sensitive inferences about the caregivers and thus, privacy invasions. This is done either by bycatch during visits (e.g., heartbeat of the caregiver) or when using the technique in one’s own home or based on inferences that can be drawn from relationships or genetic similarities. So far, there is no regulation on how to deal with incidental findings in these data [52, 53]. However, this could influence the intimacy of the care relationship. On the other hand, invasive yet precise measurements associated with SST can make end-of-life care more accessible and more reliable for informal caregivers in the future [54]. It also allows care to be provided in familiar surroundings at home or with cable-free beds. Thus, the intimacy aspect and autonomy aspect of care are Janus-faced: On the one hand, care can be planned better, and care needs are recognized more quickly. It seems to be a gain in autonomy. On the other hand, the social dimension draws attention to the fact that the autonomy of the, not only informal, caregivers, understood as relational and dependent on external enabling conditions, must be considered as well. It must be carefully examined whether SST burden or relieve the informal and formal caregivers. SST must not lead to intensified loneliness or isolation and not reduce relevant social interactions.

### Smart sensor technologies and the spiritual dimension of care

Spiritual needs and spirituality play a crucial role in palliative care [55, 56]. The definition by Christina M. Puchalski et al. summarizes what spirituality is. They define it as a “dynamic and intrinsic aspect of humanity through which persons seek ultimate meaning, purpose, and transcendence, and experience relationship to self, family, others, community, society, nature, and the significant or sacred. Spirituality is expressed through beliefs, values, traditions and practices” [55]. Both, the definition and other empirical research show that spirituality is conceptually fuzzy and a highly individual experience or practice [57, 58]. Simultaneously, the broad term may encompass a diverse range of religious and non-religious worldviews. Based on the definition by Puchalski et al., Traugott Roser et al. ask for the indications of a spiritual care need. They identify: biography, grief/despair, connection with religious community, central relationships, purpose, destiny, and ethics [59]. With this list of indications, the need for spiritual care becomes, to some degree, operationalizable. For example, the indication “biography” can show itself in the increased need for attention, a patient appearing blocked, burdened by feelings of guilt and shame, and revolving around unfinished businesses [59]. This may concern issues of guilt and forgiveness, biographical ruptures, etc., which the caregiver then addresses [59]. With this set of criteria, it may be possible to use SST in a facilitating way to identify spiritual care indications and to measure the influence of spiritual care on stress or other symptoms. However, it is important not to lose sight of the fact that SST is a limited tool to support capturing spiritual needs.

Second, until now, spiritual or pastoral care has been an intimate and interpersonal event that (ideally) offers a safe space through pastoral care confidentiality. Thus, the patient is able to express herself – knowing that the information is protected by confidentiality. The anthology of Simon Peng-Keller et al. draws attention to the fact that digital documentation practice can transform spiritual care relationships [60]. Supporting the spiritual care process with SST must take the sensitivity and privacy of this information into account. As required in other areas concerning digitized health care and precision medicine, [33] patients and patient representatives must be involved in deciding what information about them will be included in the anamnesis. Often the information is oriented to questionnaires, which are not very flexible [33]. This leads to the fact that information, which is structured as a narrative, is often not given space and thus does not offer a reference point for treatment. Information that is meaningful to a patient is likely to be missed.

## Conclusion

**Fig. 3:**
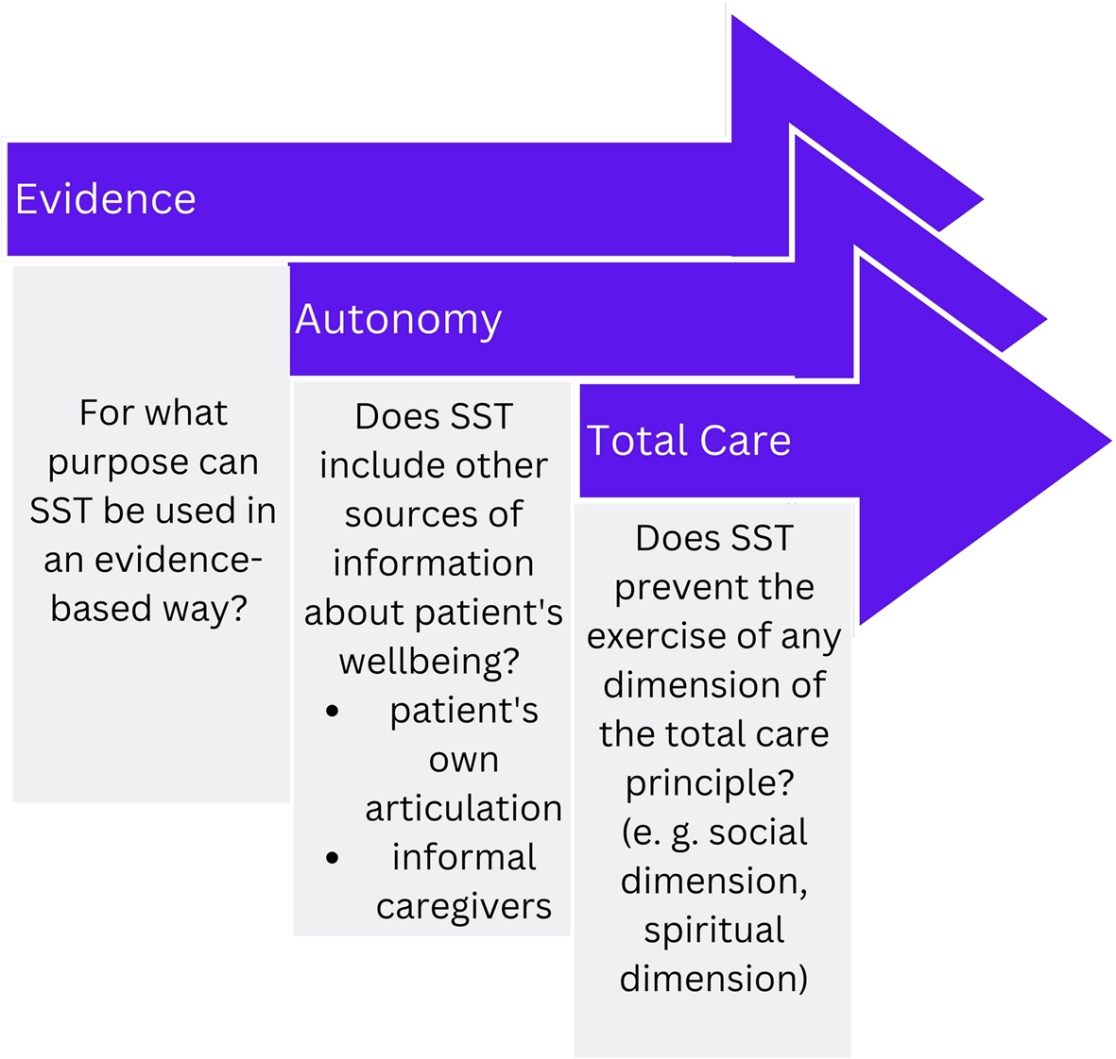
When to involve SST in palliative care? Three governance questions

Several challenges became apparent in analyzing the concepts of Total Care with respect to SST. They can be tackled by considering three questions drawn from the analysis above – cf. Fig. 3.

**For what purpose can SST be used in an evidence-based way?** For which dimension is it reasonable and adequate to use SST? [61]. SST focus on gathering quantifiable data – which leaves blank spaces regarding the four Total Care dimensions – body, mind, social, and spirit. Not every aspect relevant to the Total Care principle is measurable by SST. For instance, narrative data, life stories, personality, and values, that could give conclusions about social or spiritual suffering are likely to be left out. In addition, technology does not work equally well for every patient group. For responsible use of SST, bias and distortion must be addressed. Nevertheless, SST can support the patient’s articulation regarding some operationalizable aspects. However, the aim of supporting the patient’s articulation and patient’s autonomy will only be achieved if the SST outcomes are not set absolute. Therefore, the second question **Does SST include other sources of information about a patient’s well-being?** reminds of the necessary embedding of SST in a social context. SST must provide space for patients and their relatives to articulate all aspects of their well-being.

Finally, the third question concerns the implicit consequences of the application of SST: **Does SST prevent the exercise of any dimension of the Total Care principle?** Three examples shed light on the importance of this question. First, if SST only measure somatic parameters, it is more likely that they will become the sole criterion for medical decision-making. The other important aspects of the Total Care principle – psychological, social, and spiritual suffering – will more likely be blanked out. Therefore, the SST must, where applicable, try to include the measurement of psychological, social, and spiritual suffering as well, using evidence-based operators. However, finding measurable parameters for the psychological, social, and spiritual dimensions of care is highly subjective and prone to bias. Second, “high touch” as part of the social dimension and “high tech” (SST) are not mutually exclusive but necessarily complement each other. Third, SST must not harm the confidentiality necessary for spiritual care. All three questions are important criteria on the way to a responsible use of SST that considers the conditions and challenges of the care situation in palliative care.

## Data Availability

Not applicable. Data sharing is not applicable to this article as no datasets were generated or analysed during the current study.

## Abbreviations

BAI: Beck Anxiety Inventory
SST: smart sensor technologies
